# Evaluating the success of the tele-pathology system in governmental hospitals in Kuwait: an explanatory sequential mixed methods design

**DOI:** 10.1186/s12911-021-01567-x

**Published:** 2021-08-02

**Authors:** Ali Jasem Buabbas, Tareq Mohammad, Adel K. Ayed, Hawraa Mallah, Hamza Al-Shawaf, Abdulwahed Mohammed Khalfan

**Affiliations:** 1 Department of Community Medicine and Behavioural Sciences, Faculty of Medicine, Kuwait University, Safat 13110, P.O. Box 24923, Jabriya, Kuwait; 2 Department of Pathology, Jaber Al Ahmad Al Sabah Hospital, Ministry of Health, South Surra, Kuwait; 3 Department of Surgery, Faculty of Medicine, Kuwait University, Jabriya, Kuwait; 4 Department of Internal Medicine, Mubarak Al Kabeer Hospital, Ministry of Health, Jabriya, Kuwait; 5 Department of Health Informatics and Information Management, Allied Health Sciences, Kuwait University, Jabriya, Kuwait; 6 Department of Computing and Information Systems, The Public Authority for Applied Education and Training, Ardhiya, Kuwait

**Keywords:** Telepathology system, Evaluation, Attitude, Pathologists

## Abstract

**Background:**

Telepathology is the practice of reviewing and exchanging pathological images through telecommunication systems to obtain diagnoses remotely. Studying the factors that make such a system successful and favourable is important to ensure the merits of its implementation in clinical practice.

**Objective:**

This study aims to evaluate the success of a telepathology system from the users’ perspectives, using specific evaluation criteria, namely: system quality, information quality, technical service quality, user satisfaction, and benefits.

**Methods:**

A sequential explanatory mixed methods design was adopted in this study, which consists of two phases. Initially, a questionnaire was distributed via WhatsApp to all of the pathologists (total: 45) working at governmental hospitals in Kuwait. Followed by, semi-structured interviews with ten senior pathologists.

**Results:**

Forty pathologists responded to the questionnaire, giving an 89% response rate. There were 42.5% of the respondents aged between 35–44 years old, and 52.5% were male. The quantitative results reveal that most of the respondents were satisfied with the quality of the telepathology system with a mean of 2.6025 (Standard Deviation (SD) = 0.47176), whereas they were dissatisfied with the quality of the information with a mean of 2.4100 (SD = 1.580) and the technical support services with a mean of 2.2750 (SD = 0.99535). In addition, there was disagreement on the benefits of telepathology in clinical practice among the pathologists with a mean of 2.4667 (SD = 0.77552). The qualitative results indicate that the lack of interest in and little experience with using the system were behind the general dissatisfaction of most of the respondents. All of the interviewees were satisfied with the performance of the telepathology system and considered it successful; however, the quality of the technical support services, including training workshops, was deemed deficient.

**Conclusion:**

This study concluded that telepathology system in Kuwait is functioning well and has been successful in its implementation; however, pathologists are dissatisfied with it, mainly due to the deficient quality of the technical support services provided. In addition, the successful implementation of such advanced technologies requires careful steps to be taken on multiple levels: technical, organisational, and managerial. Recommendations were suggested.

## Background

Telepathology, a subset of telemedicine, is the practice of reviewing and exchanging pathological images through telecommunication systems to obtain diagnoses remotely. Telepathology has numerous advantages when it’s successfully applied in medical care. It brings individual, operational, and quality-of-service advantages [1, 2]. At the individual level, the shortage of pathologists has been a major problem worldwide; therefore, telepathology systems are widely used to obtain primary or secondary diagnoses. Besides, the implementation of telepathology facilitates the discussion of difficult cases among pathologists, enabling them to feel more secure in their diagnoses [1]. At the clinical level, the time needed to obtain slides is decreased, leading to an earlier diagnosis and earlier management of the patient, in turn causing the quality of services to be improved [3]. Moreover, with telepathology, there is no need to send the specimen offsite, thus helping the surgeon to make a decision and proceed with the surgery [2, 4]. Telepathology has been playing a role in consultations during surgeries, as in the case of intraoperative consultation (frozen section diagnosis). Financially, using the conventional way of transferring pathology slides can be costly and may lead to slides being damaged; however, this can be avoided by using telepathology techniques. Hospitals can also reduce costs with telepathology by reducing the number of staff required on-site [2, 5]. Nevertheless, the available literature reveals gaps with regard to the telepathology systems’ effectiveness and efficiency concerning their intended use.

On the other hand, possible drawbacks have been identified for telepathology, which are pertaining to user experience, costs and efficiency gains, and general compatibility within routine work and with other systems, such as the laboratory information system (LIS) [6]. Therefore, evaluating the telepathology system after implementation to assess the impact on practitioners and practice is considered a crucial step to prove the success, as the greater the benefits of the system, the greater the impact on clinical productivity.

Telepathology systems have been used in many hospitals worldwide, including the developing nations, despite the high cost and complexity of the information technology infrastructure required [7]. The available literature is abundant with studies of telepathology systems, with each study evaluating the system (s) based on certain aspects. Previous studies have compared the traditional method with digital ones [8], analysed users’ attitudes towards telepathology [9], examined the impact of telepathology [10], and investigated the diagnostic services provided by telepathology [11].

Investigating pathologists’ attitudes towards the use of telepathology is one of the most important factors behind a successful implementation. This would facilitate exploring the users’ satisfaction with regards to the system functionalities and its effect on clinical practice. In 2016, a study conducted on the impact of a large and decentralised telepathology network in Canada revealed the general satisfaction of the pathologists with the quality of the virtual slides and images generated by the system; however, many of the pathologists mentioned that they could not achieve a precise diagnosis with a digital slide when faced with complex cases and would rather follow the “normal” procedure [12]. In 2005, a national survey conducted in the UK investigated the use of digital imaging and telepathology in histopathology [13]. It concluded that most of the histopathologists did not routinely use or have access to a telepathology workstation. Consequently, telepathology was never used in their daily diagnostic work and was rarely used on a weekly or monthly basis. Most of the respondents identified a role for telepathology / videoconferencing in facilitating remote attendance at multidisciplinary cancer team meetings and educational seminars. They also identified a potential use in the referral of cases for expert opinions but had little interest in routine use for remote reporting [13]. In regards to the users’ need for the proper training programs, a study conducted on attitudes and practices in the Canadian pathology community observed favourable responses towards pursuing digital pathology training: 57% of the pathologists and 77% of the residents stated that they would attend informatics workshops if they were made available [14].

### Telepathology in Kuwait

The Ministry of Health (MOH) in Kuwait first adopted telepathology technology approximately ten years ago. Initially, it was piloted and used by the Kuwait Cancer Control Centre (KCCC) to seek consultations from expert pathologists at the University of Toronto in Canada. Later in 2014, the MOH purchased six telepathology scanners (Aperio Digital Pathology Scanners Model: AT2 by Leica) at a cost of about two million KD (USD 6,583,652.00), including equipment, infrastructure modification, connection, and technical support. Aperio™ is a digital slide scanning system from Leica Biosystems. It offers the ability to capture and covert histo/cyto-pathology glass slides into high quality digital images enabling local or remote onscreen viewing for diagnostic and research purposes instead of using conventional microscopy [15]. The scanners were installed in five major hospitals, namely: Mubarak Al-Kabeer Hospital, Farwaniya Hospital, Amiri Hospital, Al-Adan Hospital, and the KCCC in the Al-Sabah health region. Like every other country, Kuwait suffers from a major shortage in qualified pathologists, including anatomic pathologists. Hence, telepathology was employed to cover this shortage and improve the quality of medical care.

From the above, it can be considered that the evaluation of telepathology systems is important in order to understand the factors that make such a system successful and favourable and to ensure the merits of its implementation in clinical practice. There is a lack of studies of this kind in Kuwait and Middle East region that have deeply evaluated telepathology systems in clinical practice. This led us to conduct a study that aimed to evaluate the success of telepathology in clinical practice from pathologists’ perspectives, using specific evaluation criteria (quality of information, quality of system, quality of services, benefits), in addition to identify the current advantages and drawbacks of the telepathology system.

### The research hypothesises were

- Pathologists’ satisfaction is significantly associated with the impact of the telepathology on the quality of the information, quality of the system, and quality of technical services.
- The more the pathologists satisfied, the more they use telepathology system, the more the benefits on clinical practice
- There are significant differences with pathologists’ satisfaction (quality of information, quality of system, quality of services), according to gender, age, computer competency, and experience.

## Methodology

### Study design, population, sampling method and research setting

A sequential explanatory mixed methods design was adopted in this study, which consists of two phases. Initially, a validated questionnaire was adapted from a previous study to collect data [10]. This questionnaire (quantitative method) was distributed via WhatsApp-a smart phone messaging application- to all of the pathologists of all levels from assistant registrars (senior house officers) to consults working at governmental hospitals in Kuwait. Followed by, semi-structured interviews (qualitative method), which came to validate the quantitative findings and were conducted with senior pathologists including a head of department and a senior registrar from each hospital’s pathology department. This study design help to provide an in-depth understanding of the general attitudes of the pathologists and their satisfaction towards the impact of the telepathology system on their work, according to specific evaluative criteria. Also, thorough explanations are obtained via the interviews to explain and elaborate the trends that emerged from the quantitative findings.

### The quantitative method: questionnaire

The questionnaire was revised by an academic team: Two from faculty of medicine, one from pathology department at Ministry of Health and one from faculty of allied health sciences at Kuwait University, in order to ensure the content validity of questionnaire items are meeting the aim of this study. Thus, a pilot study was conducted with six participants to examine the suitability and readability of the questionnaire and feedback were received that required minor modifications.

A web survey was developed via Google Forms to collect data from the pathologists. The link of the questionnaire was shared in a WhatsApp group that includes all of the pathologists working in the governmental sector in Kuwait (total: 45). This was done by head of the WhatsApp group. The data collection took 4 weeks; a soft reminder was sent once week. The questionnaire aimed to evaluate the success of the telepathology system in five hospitals based on specific criteria, where Likert-scale items were used (1 = lowest or “strongly disagree” and 4 = highest or “strongly agree”).

The questionnaire consisted of eight sections, namely: (1) demographic data: this section requested information on the participant’s age, gender, nationality, place of residency training program, and specialty level; (2) background: this section focused on the user’s experience with telepathology, frequency of using the system, and computer literacy level; (3) system quality: this section questioned if the telepathology system is fast, easy to access and to navigate, makes it easy to view images, offers a wide range of functions, and communicates with other systems; (4) information quality: this section focused on whether the system produces high-quality information that is accurate, complete, relevant, and timely; (5) service quality: this section focused on technical support reliability, promptness, and dependability, including user training; (6) user satisfaction: this section focused on the user’s attitudes towards the system’s functions; (7) system usage: this section looked at how often the user used the system for clinical functions and telecommunication; and (8) the benefits of the telepathology system: this section mainly focused on whether the system improves diagnoses, reduces the time needed reading the scanned images, improves accessibility, improves the quality of care, and reduces errors.

**Fig. 1:**
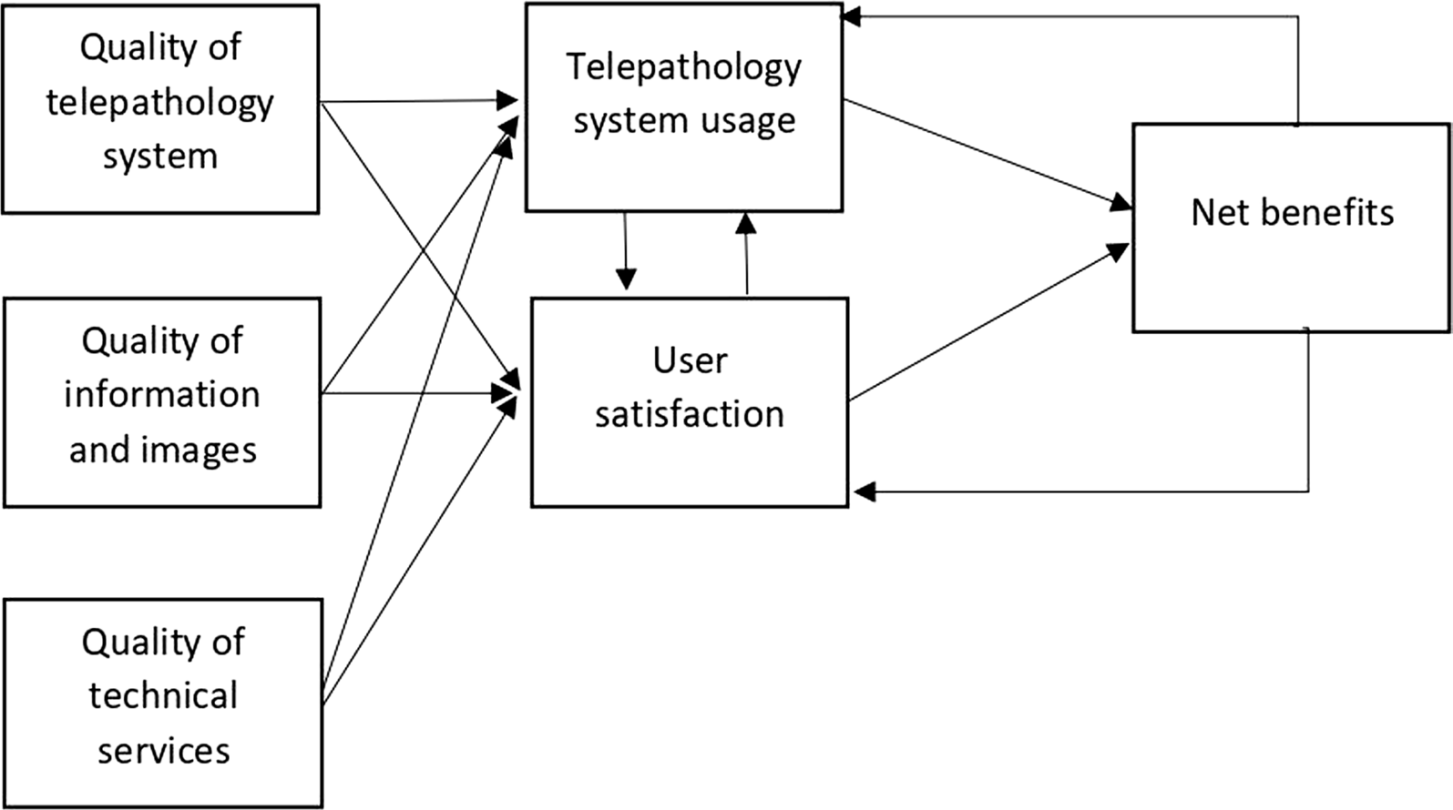
An integrated model of telepathology system success

These evaluation criteria were developed based on an integrated multidimensional model, which was constructed from the model primarily developed by Delone and McLean (Fig. 1) [16].

### The qualitative method: interviews

This qualitative part of the study aimed to discuss the main quantitative findings with leaders who had experience in using the telepathology system, in order to identify their opinions and impressions in this regard and to explore the advantages and drawbacks associated with the use of the system. In addition, it enabled the identification of suggestions to improve the system. An interview guide was developed to achieve the study’ aim, as shown in Table 1.

**Table 1:** The interview guide

What are your opinions on the quantitative findings of this study?
What do you think the reasons behind these findings are (e.g. unsuccessful implementation of the telepathology system)?
What is your satisfaction level regarding telepathology use in your department? In regard to the information quality, system, and technical support services, in addition to the benefits/advantages utilised by staff
What are the obstacles or drawbacks that hinder your department from obtaining the full advantages of telepathology? In regard to the staff, system, and technical support services
What are your suggestions to improve the telepathology practice in your department?

Ten telephone interviews based on a semi-structured approach were conducted with telepathology users from each hospital: in total, five heads of pathology departments and five senior pathologists. This approach provided a deep understanding of the telepathology use in each hospital.

The interviews were pre-arranged with the participants to be at their convenience, and a timetable was created accordingly. The interviews were conducted via telephone by the main investigator of this study, who has significant experience in this type of approach. All participants were informed about the aim of the interviews. Afterwards, open-ended questions and probes were used to guide the conversation in an appropriate way. The average duration of each interview was 20 min.

### Statistical analysis

Data management, analysis, and presentation were implemented using the Statistical Package for Social Sciences (SPSS) software, version 26.0. The questionnaire was evaluated for internal consistency and reliability, and Cronbach’s alpha values were estimated for pathologists’ perspectives by combining the Likert-scale used for specific criteria, namely: system quality, information quality, technical service quality, user satisfaction, and benefits, giving the value of 0.948, which indicates the questionnaire had a high degree of reliability (Table 2). Chi-square tests were applied to find any correlations or significant differences between the categorical variables, where *P* < 0.05 was considered significant.

**Table 2:** Internal consistency and reliability of the questionnaire items

Items	No. of items	Reliability	Means	SD
System quality	20	.867	2.6025	.47176
Information quality	10	.852	2.4100	.73058
User satisfaction	6	.692	2.4667	.77552
Use	2	.703	3.0500	.94598
Benefits	14	.855	2.2750	.69790
Service quality	14	.938	2.2750	.99535
General stability of the questionnaire	66	.948		

**Fig. 2:**
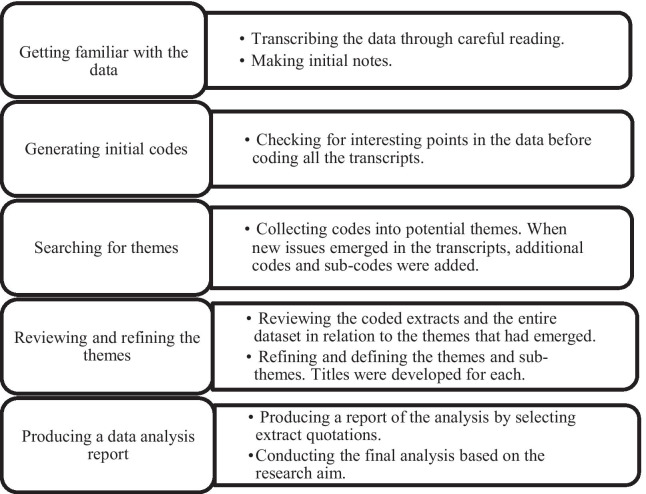
Process of the thematic analysis

To analyse the qualitative data, the interviews were transcribed verbatim by the main investigator. To ensure the accuracy of the transcripts, they were checked against the original audio-recordings. Field notes were taken during and after the interviews to ensure that all important information was documented. A qualitative approach comprising inductive thematic analysis was undertaken for the data analysis (Fig. 2) [17]. This method is simple qualitative approach, which can provide explicit results that are more understandable to the public and more attractive to the researchers due to its high flexibility of analysis [18].

The coding process was performed manually through Microsoft Word. A coding frame was developed based on the themes identified during the interviews. The coding unit of the interview was line numbers and page numbers. To ensure the reliability in the coding and the accuracy of the transcriptions, reviewing and refining the themes and sub-themes were done by the co-authors, in addition to cross-checking a random sample (n = 5). Slight differences were found and resolved during discussion meetings.

## Ethical considerations

The study was approved by the Ministry of Health’s Standing Committee for the Coordination of Health and Medical Research in the State of Kuwait (2020/1293). An informed consent form was obtained from each participant who agreed to participate in the questionnaire survey or the interviews. All methods were performed in accordance with the relevant guidelines and regulations.

## Results

Descriptive statistical analysis was used to produce the frequencies and percentages for all of the Likert-scale items in the questionnaire (1 = lowest or “strongly disagree” and 4 = highest or “strongly agree”). Accordingly, the average weighted mean was calculated, where intervals of four levels were used: first level = 1–1.74, second level = 1.75–2.49, third level = 2.5–3.24, and fourth level = 3.25–4. The first and second levels of interval indicate “disagree or strongly disagree” response, and third and fourth levels of interval indicate “agree or strongly agree” response.

### The quantitative results

Table 3 shows the demographic data of the questionnaire respondents, who represented 89% of the pathologists in Kuwait’s governmental healthcare sector. The results show that 42.5% of the respondents were in the age group of 35–44 years old, and 52.5% were male. The Kuwaiti respondents outnumbered the non-Kuwaitis. Most of the respondents were consultants and had non-Kuwaiti board. In regard to the respondents’ experience in using telepathology, the highest percentage was for those with 2–4 years’ experience.

**Table 3:** Demographic data and characteristics of the respondents

Demographic variable	Frequency	Percentage
**Age**		
25–34 years old	10	25
35–44 years old	17	42.5
45–54 years old	8	20
55 and above	5	12.5
**Gender**		
Male	21	52.5
Female	19	47.5
**Nationality**		
Kuwaiti	22	55
Non-Kuwaiti	18	45
**Specialty level**		
Assistant registrar	2	5
Registrar	7	17.5
Senior registrar	9	22.5
Specialist	10	25
Consultant	12	30
**Place of residency board**		
Kuwaiti board	14	35
Other board	26	65
Less than 1 year	4	10
**Experience in using telepathology**		
1–2 years	6	15
2–4 years	12	30
4–6 years	10	25
More than 6 years	8	20
Literate	11	27.5
**Computer skills**		
Competent	20	50
Proficient	9	22.5
Several times a week	6	15
**How often do pathologists use telepathology?**		
About once a week	5	12.5
About 2–3 times a month	10	25
About 3–4 times a year	14	35
About once a year	2	5
Less than once a year	10	25

### Quality of the system

The results show that the pathologists agreed that the telepathology system was of good quality, with a mean of 2.6025 (SD = 0.47176) on the four-point Likert scale. The mean fell within the third level of the intervals.

The Chi-square tests showed that there were statistically significant associations between the quality of the system and certain demographic factors, in which female pathologists (*P* = 0.027) and those with strong computer skills (*P* = 0.049) were more likely to perceive the quality of the system as high.

### Quality of the information

The mean of 2.4100 (SD = 1.580) on the four-point Likert scale indicates that the pathologists disagreed that the quality of the information provided by the telepathology system was good. The mean fell within the second level of the intervals.

The Chi-square tests showed that there were significant associations between the information quality and certain demographic factors, where female pathologists (*P* = 0.035), consultants (*P* = 0.034), those who had non-Kuwaiti board (*P* = 0.050), and those who were the most frequent users of the system (*P* = 0.010) were more satisfied than others in regard to the quality of the information provided by the telepathology system.

### Quality of the technical support services

Regarding the quality of the technical support services, the mean was 2.2750 (SD = 0.99535) on the four-point Likert scale, revealing that the pathologists generally disagreed that the quality of these services was good. The mean fell within the second level of the intervals.

The Chi-square tests showed that there were significant associations between the quality of the technical support services and having more than six years’ experience in using telepathology (*P* = 0.017) and having strong computer skills (*P* = 0.014). Pathologists in these categories were more likely to agree that the quality of the technical support services for the telepathology system was good.

### User satisfaction

The results reveal that the pathologists were dissatisfied with using the telepathology system, with a mean of 2.4667 (SD = 0.77552) on the four-point Likert scale, which fell within the second level of the intervals.

**Fig. 3:**
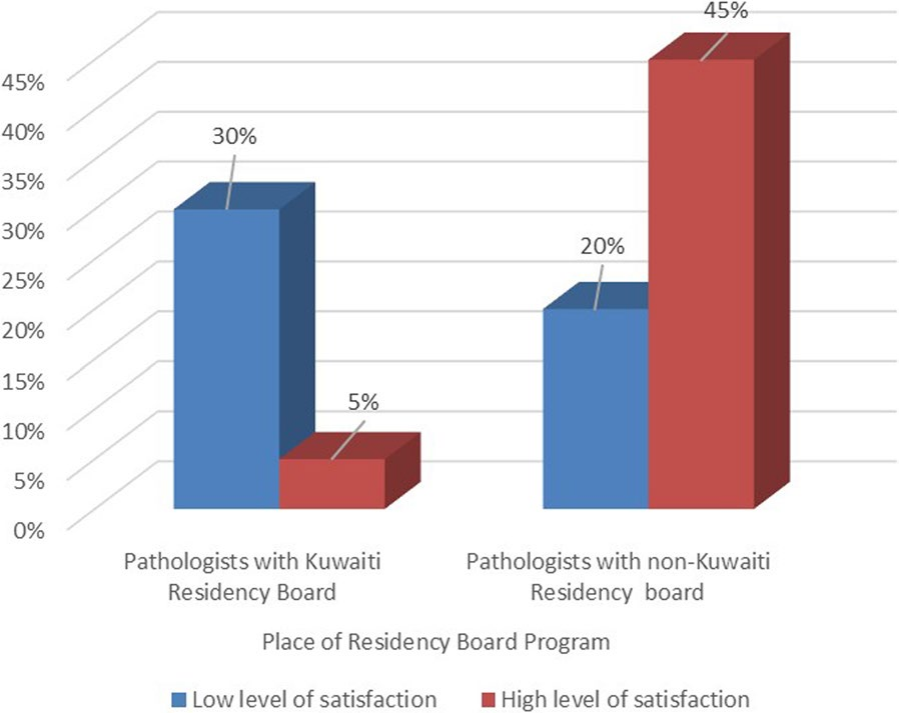
The association between the pathologists’ satisfaction and place of residency board

**Fig. 4:**
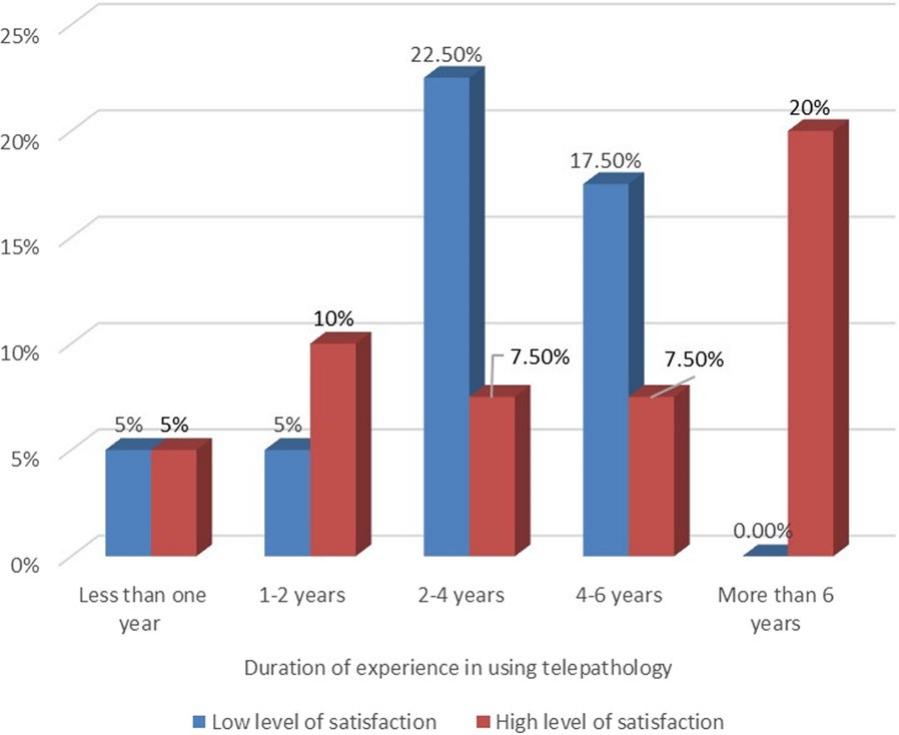
The association between the pathologists’ satisfaction and experience in using telepathology systems

The Chi-square test showed that there were significant associations between user satisfaction and certain demographics, where pathologists with non-Kuwaiti board (*P* = 0.001) and those who had more than six years’ experience in using telepathology (*P* = 0.010) were more satisfied than others (Figs. 3 and 4).

### Telepathology usage

The results showed a mean of 3.0500 (SD = 0.94598) for telepathology usage on the five-point Likert scale, which indicates that most of the pathologists were using the telepathology system on an “occasional” basis. This fell within the moderate level of the intervals.

**Fig. 5:**
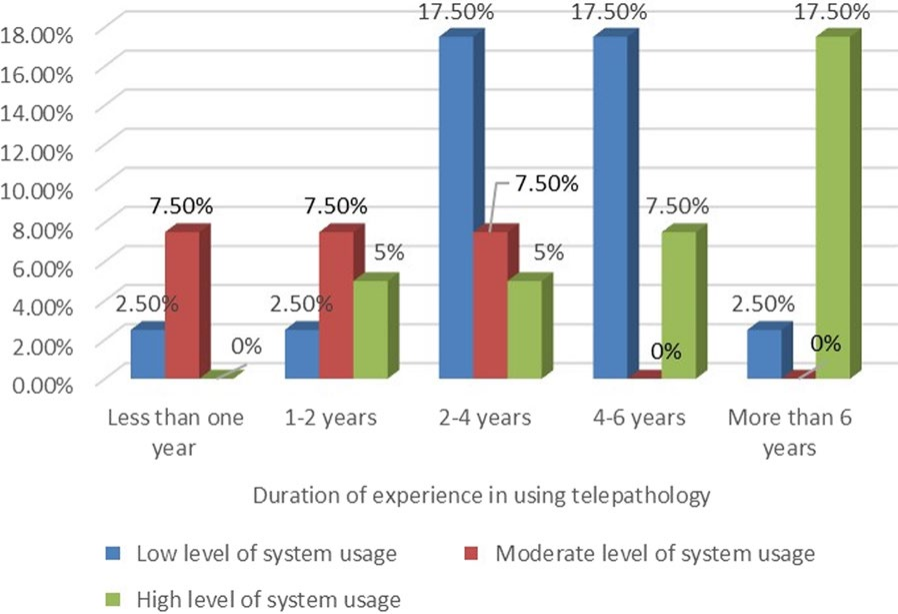
The association between the pathologists’ use of telepathology and experience in using it

The Chi-square tests showed that there were significant associations between system usage and certain demographics, including age, gender, years of experience in using such systems, and frequency of use. The results show that the male pathologists (*P* = 0.014), those in the middle age group (35–44 yrs.) (*P* = 0.015), those with more than six years’ experience in telepathology (*P* = 0.001) (Fig. 5), and those who used the telepathology system several times a week (*P* = 0.004) used telepathology more than others, in order to perform work functions and to obtain second opinions.

### Benefits of telepathology

The results show a mean of 2.2750 (SD = 0.69790) on the four-point Likert scale regarding the benefits of telepathology. This fell within the second level of the intervals, which indicate that most of the respondents disagreed with the telepathology benefits on clinical practice.

The Chi-square tests showed that there were significant associations between the benefits of telepathology and certain demographics, in which the pathologists with non-Kuwaiti board (*P* = 0.004), those with more than six years’ experience in using telepathology (*P* = 0.001), and those who had strong computer skills (*P* = 0.049) were more positive in rating and agree with the benefits of using the telepathology system.

#### The qualitative results

### Demographic data

Most of the interviewees were male (n = 5) and in their 40 s; two were female and in their late 30 s; and the remaining three were male in their 50 s or older. All of the interviewees had non-Kuwaiti boards, except the two females, who had Kuwaiti board. Four themes emerged from the data: Overall opinions on the quantitative findings of the study, pathologists’ satisfaction with the telepathology use, obstacles that hinder from obtaining the full advantages of telepathology, and the suggestions to improve the system.

### Overall opinions on the quantitative findings

All of the participants stated that they were not surprised by the findings, and they provided several reasons for them. One of the directors stated: “*We expected these results, and we know the reasons behind the dissatisfaction of the staff regarding telepathology use*”.

All of the participants were agreed that the Aperio telepathology system is a high-quality system, but “*staff resistance*” "*a lack of interest and an unwilling attitude”*, regardless of gender and age, to using the system had made them lower their estimations of the advantages of its use. Common reasons were identified by the participants, which were: a lack of hands-on training courses, a lack of awareness of telepathology’s advantages, using the system requiring more effort and time, and using it not being compulsory. One responded: “*I am sure the reason is that pathologists use the Aperio system only sometimes, not on a frequent basis, which makes the staff unfamiliar with the system’s usage and benefits*”. Most of the participants (eight out of ten) said that the technologists were more involved in scanning the glass slides and adjusting the angles of the pathological images, while the role of the pathologists was to study the images and write reports.

All of the participants declared that the current telepathology is very useful, but persistence is needed by all to utilise the advantageous functionalities of the system. One participant enthusiastically said: “*Persistence is required in order to use the new system… The Aperio system can be used for multiple purposes, beside remote consultations. The high-resolution images provided with high zooming capabilities can be used for teaching residents (students) and presenting the images easily to colleagues at conferences*”.

### Pathologists’ satisfaction with the quality of the system, information, services, and benefits

The results reveal that all of the participants had no complaints about the system itself, including the user interface and the quality of the information provided. They described it as the best system for practising pathology at a distance and as a well-known system worldwide. One of the department heads stated: “*Using telepathology is very advantageous, so 85% is the utilisation rate of Aperio in our hospital*”.

On the contrary, regarding the quality of services and benefits, variations in responses were reported among the interviewed pathologists. Five out of the ten participants complained about the reluctance of the company to provide prompt technical support. “*The response from IT support at the company is not at a satisfactory level*”. Some of the participants commented that the company was not providing maintenance or IT support equally to all hospitals.

In regard to the training courses, most of the participants (seven out of ten) commented that the programme was not hands-on. Additionally, three of them stated that the training was not offered on a flexible timetable. A senior pathologist who had attended a training course commented: “*Some of the pathology staff did not know what digital pathology was, not only as a term but also as a clinical tool – how to use it and for what purpose, as well as the benefits and outcomes.*” Another commented: “*due to the absence of flexible training programmes, the system is not used frequently, as staff do not utilise its functionalities and capabilities much and are satisfied with conventional pathology practice*”.

### Obstacles that hinder pathologists in using the system

The interviews explored the obstacles that hinder the pathologists in fully exploiting the telepathology system. All of the participants reported the lack of integration of the Aperio system with LIS and the hospital information system (HIS), so the pathologists had to use more than one computer system for each case. Furthermore, the storage capacity was considered insufficient, meaning images had to be deleted from time to time. This problem was reported by the majority of the participants (n = 8). One commented: “*We need the system to be able to store more images, as the capacity of the storage is getting full, and this drawback will prevent us from utilizing it*.” Another said: “*The telepathology system is not an archiving system, so if it connected to the LIS and HIS, the storage capacity would be improved; therefore, we archive only the important images (e.g. cancer cases). For other images, we save the slides physically – the traditional way of slide filing*”.

It was reported by one of the department heads that the location of the scanner hindered staff from routinely using the system, as it was located far away from their offices.

The interview results included suggestions from pathologists’ perspective to improve telepathology implementation in hospitals (Table 4):

**Table 4:** Suggestions from pathologists’ perspective to improve telepathology implementation in hospitals

Suggestion (1)	The best way to practise pathology remotely is via computer systems, and smart technology applications can play a significant role in practising pathology on the go. A notification service could alert users via email, SMS, or other massaging services such as WhatsApp when new cases are received
Suggestion (2)	Integrating the telepathology system with LISs and HISs would improve the storage capacity of the system and the sharing of information between hospitals. A senior pathologist reported: “*Recently, we asked for hard drive expansion to provide more storage capacity for our Aperio*”
Suggestion (3)	It is very important to consider ergonomic factors to make the environment suitable for the user to use the technology, such as location, lighting, workflow, etc
Suggestion (4)	Awareness sessions are required alongside hands-on training courses to show the usefulness of the telepathology system in practice
Suggestion (5)	Giving the pathologists the authority to access Aperio from their homes would allow them to receive more of telepathology’s benefits

## Discussion

This study has evaluated the success and impact of a telepathology system in pathology practice. The findings are twofold: the quantitative findings reveal that users were dissatisfied with the telepathology system in numerous aspects, while the qualitative findings reveal the reasons for this dissatisfaction as well as the improvements required to make the best use of the telepathology system.

### Pathologists’ opinions on the quality of the information

The study revealed that the majority of the pathologists were not satisfied with the quality of the information provided by the system. The interview results explain the reasons behind these findings, including the reluctance of pathologists to use the system frequently. This could be not due to the system or the quality of its information but to the personal factors of the users, such as a resistance to technology or new systems and a lack of interest, knowledge, and skills in using the system efficiently. In line with this, the interview results confirm the lack of workshops to increase awareness of the system benefits and encourage users to use the system and utilise its functionalities in the proper way. So, the less a pathologist uses the system, the less knowledgeable they will be with regard to its usefulness, and the less likely they will develop skills in using the system. This finding is consistent with a study conducted in Ireland, suggesting that the greater the amount of tissue viewed by a pathologist, the more comfortable they are using the system and the more likely they are to make a correct diagnosis [19].

Interestingly, significant associations were found between certain demographic factors and the information quality: females, consultants, pathologists of non- Kuwaiti board, and those with more than six years of experience in telepathology were more satisfied with the quality of the information than others were. The possible reason for this satisfaction could be that those pathologists used the system often during their residency years, so they were more familiar with using the system, thus finding it easier and of a good quality. This was supported by the interview results, which confirm that the more a pathologist uses the system, the more they become familiar with its functionalities and the more useful they find it in clinical practice.

### Pathologists’ opinions on the quality of the technical support services

It was found that the majority of the pathologists were neither satisfied with the technical support services available, nor the training provided, which leads to less usage of the system and less benefits. These findings indicate that there was no cooperation between stakeholders, which is one of the most significant factors behind successful implementation of any system. Moreover, many of the pathologists reported the lack of training and knowledge provided by the company (Aperio). These findings are consistent with the qualitative interview results, as well as with previous studies [13, 20], where lack of technical support and suitable training for users are considered barriers to accept the new system.

### Pathologists’ opinions on the quality of the telepathology system

The findings of the study show that the respondents were generally happy and satisfied with the quality of the system. The pathologists reported that the system was easy to use and control and provided quick responses. The system could be easily accessed through their work or even home computers. These findings indicate the happier the users about the quality of the system, the more they would use it to improve the work. A study from USA has successful validated the remote access and use of telepathology system during the Covid-19 pandemic and acknowledging its usability and usefulness in pathology practice [21]. The interview results support these findings; however, some pathologists had complained that they did not have the authority to access the telepathology system from home to obtain the full advantages of the system. This is indicated that there was an unequal technical support that might not be able to provide all attention to all clients to enlighten them with telepathology advantages.

### Opinions on the impact of the telepathology system

#### User satisfaction

The quantitative findings revealed the unwilling attitudes of the users; most of the pathologists did not like using the telepathology system in clinical practice, preferring the conventional way of using microscopes. Thus, this has indicated that the less the pathologists satisfied, the less they use telepathology system, the less the benefits would be on clinical practice. This is consistent with studies conducted in the UK and Canada [11, 13], in which there was resistance to the use of telepathology among clinical staff and a preference for the conventional methods. Previous studies have reported the same pattern and have attributed such results to deficient training and the limitations of the telepathology company [13]; however, they could also be attributed to personal factors, such as the user’s unwillingness to learn about the system and their tendency to prefer traditional methods. This resistance could be explained by legal concerns, technological barriers, scepticism, and a lack of trust from the user’s side [22].

The present study further revealed that demographic factors had a significant influence on the user’s satisfaction, where pathologists with non-Kuwaiti board and those with more than six years’ experience using telepathology were more satisfied with using the system. The interview results support these findings: all of the interviewees were totally satisfied with the advantages offered by the system, but half of them were dissatisfied with the quality of the technical support provided by Aperio, which was said to be reluctant to respond promptly to some of their requirements. In addition, the quality of the workshops provided was considered unsatisfactory.

### Benefits of telepathology

In regard to the benefits provided by the telepathology system, the findings reveal that using the system was not considered beneficial by the majority of the pathologists. This could be interpreted that limited use of telepathology system would give this outcome of no touchable benefits on routine work productivity. A study from USA has confirmed the feasibility on telepathology’ usability during the emergency case, which requires the daily use of the system to realise its impact on clinical work [21].

Most of the pathologists found that the system improved the quality of diagnoses; however, they said that it did not reduce medical errors or decrease the time needed for a surgical procedure, nor how quickly a preliminary report could be produced. It seems that in most hospitals in Kuwait with exception of Al-Sabah tertiary hospital and Adan general hospital, the system is not routinely used for intraoperative consultations. This finding contradicts those of previous studies, which have suggested that telepathology does have an influence in reducing the time needed for two-stage surgical procedures [11, 12]. This was also confirmed by the interviews of this study, wherein the participants of the pathologists acknowledged the advantages of the telepathology system over conventional methods in regard to the reduced time spent on communication and improve work productivity, which had been confirmed in previous studies [23, 24]. The quantitative findings of this study reveal significant associations between demographic factors and the perception of the telepathology system as beneficial, where pathologists with non-Kuwaiti board, those with more than six years’ experience in using telepathology, and those with strong computer skills found the system more beneficial compared to other pathologists. It seems that active users of the system were competent in computers and thus received the full benefits of the system, influencing their intention to continue utilising these advantages. This was reported in a previous study [25] that found pathologists’ experience with technology would facilitate reviewing the digital images more efficiently, also this was found in previous evaluative studies on picture archiving and communication system [24].

## Limitations

This study had limitations: (1) the questionnaire was very comprehensive in assessing the major evaluation criteria, which made it lengthy to complete, and this could have caused the respondents to feel bored and offer different responses; (2) the responses to the self-reported questionnaire could have been biased in underestimating the system’ assessment. It was found that most of the pathologists were not interested in using the system, which caused them to use it less frequently and thus accumulate less experience with the system – this could have influenced them to underestimate the telepathology system; and (3) the system was evaluated based on specific criteria. Therefore, using other evaluation models comprising different variables could have produced different results.

## Conclusion

The successful implementation of telepathology has had a great impact on the clinical field in many countries, but this does not mean that the systems are flawless. This in-depth study concluded that the telepathology system in Kuwait is functioning well and has been successful in its implementation. However, pathologists are dissatisfied with some aspects of it, mainly due to the deficient quality of the technical support services provided, which could lead to less willing to use system. In addition, the successful implementation of such advanced technologies requires careful steps to be taken on multiple levels: technical, organisation, and managerial. Overcoming obstacles is crucial to achieving the intended outcomes of telepathology systems. Accordingly, the following recommendations are provided.

## Clinical implications and recommendations

The findings of this study provide a guide for policy makers, hospital managers, and heads of the pathology departments to help them monitoring the current use of the telepathology and to employ the suggested recommendations to ensure its merit and worth in clinical practice.

The main recommendation is for a central telepathology unit to be established that is responsible for the following:

- Monitoring the performance of the telepathology system, including users’ requirements, such as providing the authority for users to access the system from home.
- Developing guidelines to define the roles of pathologists and technologists in regard to telepathology use. This should also cover the security of the system and the confidentiality of patient information.
- Upgrading the system, including integration with LISs and HISs and the adoption of smart technology.
- Arranging staff training courses that are hands-on and take a step-by-step approach to cover all the functionalities, making the trainee aware of the advantages and benefits of telepathology for clinical practice, patients, and healthcare organisations.
- Working as a liaison with the Aperio technical support team to facilitate communications and to expedite the process for support.
- Providing health informatics consultants to help users to use the system and to report any technical or managerial problems relating to the system within the work environment.

This unit should be staffed by pathologists and technologists who have the ability to influence others to adopt, accept, and make full use of this technology in practice, as the willingness of end users is a key factor in the success of telepathology. In addition, specialists with health informatics backgrounds would certainly help to support this practice and could work as liaisons between the telepathology system and pathology departments.

## Data Availability

The datasets used and/or analysed during the current study are available from the corresponding author on reasonable request.

## Abbreviations

HIS: Hospital information system:
KCCC: Kuwait cancer control centre
LIS: Laboratory information system
MOH: Ministry of health
SPSS: Statistical package for social sciences
